# Privacy Concerns About Health Information Disclosure in Mobile Health: Questionnaire Study Investigating the Moderation Effect of Social Support

**DOI:** 10.2196/19594

**Published:** 2021-02-08

**Authors:** Yuanyuan Dang, Shanshan Guo, Xitong Guo, Mohan Wang, Kexin Xie

**Affiliations:** 1 School of Business Administration South China University of Technology Guangzhou China; 2 School of Business and Management Shanghai International Studies University Shanghai China; 3 School of Management Harbin Institute of Technology Harbin China

**Keywords:** mobile health, privacy concern, disclosure benefit, health information disclosure intention

## Abstract

**Background:**

Mobile health (mHealth) provides a new opportunity for disease prediction and patient health self-management. However, privacy problems in mHealth have drawn significant attention to patients’ online health information disclosure and to the possibility that privacy concerns may hinder mHealth development.

**Objective:**

Privacy calculus theory (PCT) has been widely used to understand personal information disclosure behaviors with the basic assumption of a rational and linear decision-making process. However, cognitive behavior processes are complex and mutual. In an attempt to gain a fuller understanding of information disclosure behavior, we further optimize a PCT-based information disclosure model by identifying the mutual relationship between costs (privacy concerns) and benefits. Social support, which has been proven to be a distinct and significant disclosure benefit of mHealth, was chosen as the representative benefit of information disclosure.

**Methods:**

We examine a structural equation model that incorporates privacy concerns, health information disclosure intention in mHealth, and social support from mHealth, all at the individual level.

**Results:**

A validated questionnaire was completed by 253 randomly selected participants. The result indicated that perceived health information sensitivity positively enhances patients’ privacy concern (beta path coefficient 0.505, *P*<.001), and higher privacy concern levels will decrease their health information disclosure intention (beta path coefficient –0.338, *P*<.001). Various individual characteristics influence perceived health information sensitivity in different ways. One dimension of social support, informational support, negatively moderates the effect of the relationship between perceived health information sensitivity and privacy concerns (beta path coefficient –0.171, *P*=.092) and the effect of the relationship between privacy concerns and health information disclosure intention (beta path coefficient –0.105, *P*=.092). However, another dimension, emotional support, has no direct moderation effect on the relationship between privacy concerns and health information disclosure intention.

**Conclusions:**

The results indicate that social support can be regarded as a disutility reducer. That is, on the one hand, it reduces patients’ privacy concerns; on the other hand, it also reduces the negative impact of privacy concerns on information disclosure intention. Moreover, the moderation effect of social support is partially supported. Informational support, one dimension of social support, is significant (beta path coefficient –0.171, *P*=.092), while the other dimension, emotional support, is not significant (beta path coefficient –0.137, *P*=.146), in mHealth. Furthermore, the results are different among patients with different individual characteristics. This study also provides specific theoretical and practical implications to enhance the development of mHealth.

## Introduction

### Background

Mobile health (mHealth), which is a new type of medical service supported by the internet, medical sensors, wireless devices, and information communication technology [1], is snowballing and adding value to health care activities. The global mHealth market is projected to grow at a rate of 36.5% between 2016 and 2022, and would ultimately reach a size of US$ 22.31 billion by 2022 [2]. Through mHealth technology (e.g., mobile apps and wearable devices), health care-related information, knowledge, and consultations can be delivered to patients at any time, which is helpful for disease prediction and self-management behaviors [3].

Although mHealth offers a great potential advantage for improving public wellness, there are still some barriers (such as insufficient patient information disclosure and untimely information upload) that prevent it from being fully used. Some prior studies have worked on these problems and pointed out the factors which may lead to the barriers [4], such as unreasonable IT design [5], insufficient incentive mechanism [6], and unreliable health advice [7]. Among the factors, privacy concerns, which stem from the anxiety that personal information may be used without permission, are proposed to be the biggest barrier related to health information disclosure behaviors [8-13].

### Theoretical Foundations

Privacy calculus theory (PCT) adopts an economics perspective to gain an understanding of patients’ decisions about health information disclosure [14,15]. According to PCT, consumers conduct a cost–benefit analysis when deciding to disclose personal information in a digital context [16,17]. PCT proposes that patients are rational and make decisions by weighing costs and benefits. If the cost is higher than the benefit, patients usually choose to avoid the risk; otherwise, they will disclose personal information online. PCT can be calculated using the formula U (X) = benefit - cost or UD = F (B, C), where UD is the total calculation utility of decision D.

In mHealth, decision D is whether the patient chooses to disclose personal health information. F is a functional form; C is the opposite of vector B and includes elements that have a negative impact on UD, such as privacy concerns and information leaks. B represents the benefits arising from health information disclosure, such as obtaining necessary medical advice and useful treatment. B enhances the utility of decision D.

Social support, which is exchanged through social connections and interpersonal contacts, is treated as one of the most important benefits of online health activities [18,19]. According to the social support theory, supportive interactions, along with social support itself, protect patients from the health consequences of stress, increase adherence to treatments, and enhance recovery [20,21]. When patients are under health pressure, they need 3 types of social support from others: emotional support, informational support, and substantial support [22]. In mHealth, patients can share their medical experiences and obtain social support from other users [23]. Studies have indicated that of the 3 types of social support, mHealth is especially capable of providing informational support (such as recommendations and suggestions from similar patients or physicians) and emotional support (expressions of emotional care, such as understanding, caring, compassion, and sympathy) [24,25]. While mHealth does offer other benefits such as entertainment or financial returns (eg, gifts or discount vouchers) [26], within the PCT formula, social support is the strongest potential benefit related to patients’ information disclosure decisions in mHealth.

Individuals with different characteristics (such as personality and experience) could have different perceptions of the costs and benefits of health information disclosure. Personality may play a role by directly affecting the risk/cost perception of disclosing private information. For example, for patients who are strong in self-protection and self-sensing, privacy leaks may be crucial concerns [27]. By contrast, patients who prefer personalized services would obtain greater perceived benefits from the disclosure of personal information [28], which may moderate their attitudes toward privacy concerns and information disclosure.

### Objectives

The basic assumption of PCT is that privacy-related decision making is a rational and linear proceeding. However, in real life, people’s cognitive behavior processes are complex and mutual. Information disclosure benefits may have a nonlinear relationship with privacy concerns, and this relationship may affect patients’ health information disclosure intention or even influence their privacy concerns. That is, when facing different levels of benefits (eg, social support), patients’ privacy concerns may have different negative impacts on their information disclosure behaviors. To better understand patients’ mHealth usage and their privacy concerns regarding health information disclosure behaviors, this study takes a PCT-based approach to optimizing the cognitive behavior of patients by identifying the mutual relationship between costs (privacy concerns) and benefits (social support). Therefore, we study the interaction effect of privacy concerns and social support on individual patients’ willingness to disclose health information in mHealth.

### Hypotheses and Research Model

#### Privacy Concern and Health Information Disclosure Intention

Previous studies have accumulated considerable knowledge about the relationship between users’ privacy concerns and the ultimate use of their personal information. [29]. In the e-commerce field, Yoo et al [30] argued that privacy concern is inversely related to consumers’ engagement behaviors. In the context of health care activities, Tang et al [31] focused on the negative impacts of privacy concern on the adoption of electronic medical records, stressing that users’ privacy concerns are one of the main barriers to system adoption. Kam and Chismar [32] argued that disclosure of health information is influenced by 3 factors: patients’ perceived privacy, environmental complexity, and the value of personal information content and feedback. Simon et al [33] also pointed out that privacy concern is one of the main factors affecting users’ attitudes toward the exchange of health information. Thus, based on the literature, we hypothesize that:

H1: Privacy concern has a negative impact on patients’ intention to disclose health information.

#### Individual Characteristics and Privacy Concern

Perceived health information sensitivity [34,35] is the individual characteristic evaluated for its relationship with health information disclosure, and it reflects user preferences for information provision. For instance, Wang and Petrison [36] pointed out that when individuals give and obtain information from others, the perceived information sensitivity of the givers affects the probability that the receivers will actually obtain the information. We believe that patients with higher perceived health information sensitivity will make hasty judgments about disclosing health information. Thus, we hypothesize that:

H2: Higher perceived health information sensitivity is positively associated with privacy concern.

It is widely believed that information sensitivity varies from one individual to another [37]. Some studies have pointed out that perceived information sensitivity is mainly related to individual physiological characteristics and external characteristics [35,38]. Physiological characteristics can include disposition, social demographic variables, and personality [39-41]. Compared with dispositions and social demographic variables, personality is more likely to change over time [42]. Therefore, in the rapidly developing mHealth research context, we opt to use personality to represent personal physiological characteristics. Specifically, we use Goldberg’s 5 personality traits (extroversion, agreeableness, emotional instability, conscientiousness, and intellect), which have been widely applied in online behavior studies [43,44], to measure personality [45].

Extroversion refers to an individual’s attitude toward others, including traits such as talkativeness, boldness, determination, and sociability [46]. Extroverts are satisfied by communication with others, and they need more communication than introverts. In order to adapt to their higher communication needs, extroverts generally have lower information sensitivity [47]. Therefore, patients with high extroversion may be highly likely to share health information in an mHealth context.

Agreeableness refers to the traits associated with an individual’s affinity and co-production with their communities [48]. An agreeable person can be described as warm, harmonious, and cooperative. Empirical studies point out that people who are agreeable are more upset by deviant behavior [49]. Therefore, people who show agreeableness may be more sensitive about health information collection behaviors in mHealth. That is, patients with high agreeableness are more likely to have higher health information sensitivity than others.

Emotional instability is characteristic of a person who is often more prone than others to anxiety, depression, stress, and vulnerability [50]. In general, such a person may experience more negative emotions and is likely to be wary of dangerous situations [51]. Patients with higher emotional instability are inclined to be more nervous about disclosing their health information, as they perceive that it may put them in dangerous situations.

Conscientious people are considered vigilant and insightful, reflecting their degree of determination and expectations [52]. Academic terms used to describe conscientious people include organized, reliable, thorough, and rigorous. DeNeve and Cooper [53] argue that conscientious people are more likely to be rigorous in their work and life. Conscientious people orient their behavior toward the prevention of risk and loss [54]. Because health information is important and may cause damage to life when it is violated, conscientious patients are more sensitive to the exposure of medical and health information.

Intellectual people can be described as being imaginative, talented, wise, and logical [50,55]. Research shows that people with intellect-oriented personality traits can better reduce the risks they face and have little concern about information sensitivity [56]. Such people can reasonably analyze the current situations and take appropriate measures to avoid loss. Similarly, when using mHealth, intellectual patients are likely to be more confident in handling personal health information and thus are less sensitive to health information.

Therefore, we hypothesize that:

H3a: Extroversion has a negative impact on perceived health information sensitivity.

H3b: Agreeableness has a positive impact on perceived health information sensitivity.

H3c: Emotional instability has a positive impact on perceived health information sensitivity.

H3d: Conscientiousness has a positive impact on perceived health information sensitivity.

H3e: Intellect has a negative impact on perceived health information sensitivity.

In terms of individual external characteristics, the external environment (eg, life, economic, cultural, and legal context) influences individuals’ sensitivity to privacy information [39]. In addition, many scholars have pointed out that past adverse events can affect people’s information sensitivity [34,57]. In practice, adverse events also reflect the different influences of the external environment on individuals’ characteristics. Moreover, in the novel environment of mHealth, patients who have experienced privacy invasion will be better able to make judgments and construct their sensitivity level regarding health information. Therefore, past experience of privacy invasion is used to explore the relationship between individual external characteristics and perceived health information sensitivity.

To create a hypothesis along these lines, we consider that, as per Pavlou and Gefen [58], previous negative experiences create a lasting effect of increased sensitivity in risk evaluation. Consequently, these users will be anxious that similar services may have a high possibility of violating their psychological contracts. Therefore, experiences of privacy invasions will increase their sense of privacy sensitivity. In other words, the more an individual has been subjected to privacy invasion in the past, the higher their sensitivity toward privacy invasion would be now. Thus, we hypothesize that:

H4: Past experience of privacy invasion is positively associated with perceived health information sensitivity.

#### The Moderation Effect of Social Support

The social exchange theory emphasizes that a person will benefit other persons when he/she receives support from the social network [18]. In mHealth, both informational and emotional support can be provided through online consultations, patient groups, and shared articles.

Informational support, such as advice and assistance, can provide problem-centered solutions that allow the recipient to try to take action against the illness. In the mHealth context, informational support benefits users through professional information services, such as doctor advice or professional health care knowledge. Beneficiaries of such support are also more likely to exchange and share information and experiences in order to help each other. Extensive studies have affirmed the decisive role of informational support [59]. Wilson et al [60] pointed out that perceived informational support makes patients feel healthy.

Meanwhile, emotional support, such as compassion and concern, can help patients manage the negative effects associated with illness [61,62]. This emotional support is also available in the mHealth context, where a consultation with a doctor or communication with other patient may provide emotional encouragement and support in addition to informational support. A user who receives emotional support from the doctors or other patients will then feel obliged to reciprocate. This interactive mechanism encourages users to share their information and knowledge. Liang et al [63] proposed that both informational and emotional support have a positive impact on users’ personal information disclosure.

Overall, then, social support helps patients in mHealth through mutual aid and strengthening their capability for self-assistance. Some empirical research has found that even though many users are extremely sensitive to privacy when facing material or emotional rewards, they would nonetheless overlook their privacy concerns and disclose personal information in exchange for rewards [26]. In other words, individuals’ decisions about disclosure are not based on rational and linear trade-offs, but are irrational. Specifically, when a patient chooses to use mHealth, they are likely to fully accept the benefits of mHealth care (such as informational and emotional support), which weakens their privacy concerns about information disclosure. When patients receive more social support, although their privacy concerns are not reduced, the influence of privacy concerns on their intention to disclose health information will be reduced. That is, social support and privacy concerns have a reverse interaction effect on patients’ intention to disclose health information. Specifically, it can be assumed that:

H5m: Informational support has a negative moderation effect on privacy concerns and online health information disclosure intention.

H5n: Emotional support has a negative moderation effect on privacy concerns and online health information disclosure intention.

Research on the effect of individual characteristics on privacy concerns shows that it changes in different situations [64]. Different potential benefits may have different effects on individual characteristics and privacy concerns [12]. For example, studies have shown that a patient with a personality highly characterized by extroversion or intellect may be much more curious about knowledge and eager to be recognized [39], so informational support may reduce his/her privacy concerns. By contrast, patients with high emotional instability or conscientiousness may find emotional support more important in the mHealth context [65], and it may eliminate their privacy concerns about information disclosure. Moreover, social support is proven to help to assuage privacy concerns of those who experience privacy invasion [66]. Therefore, social support may have a moderation effect on the relationship between individual characteristics and privacy concerns. That is, it can be assumed that social support and individual characteristics have a reverse interaction with patients’ privacy concerns.

H6m: Informational support has a negative moderation effect on the relationship between perceived health information sensitivity and privacy concerns.

H6n: Emotional support has a negative moderation effect on the relationship between perceived health information sensitivity and privacy concerns.

**Figure 1:**
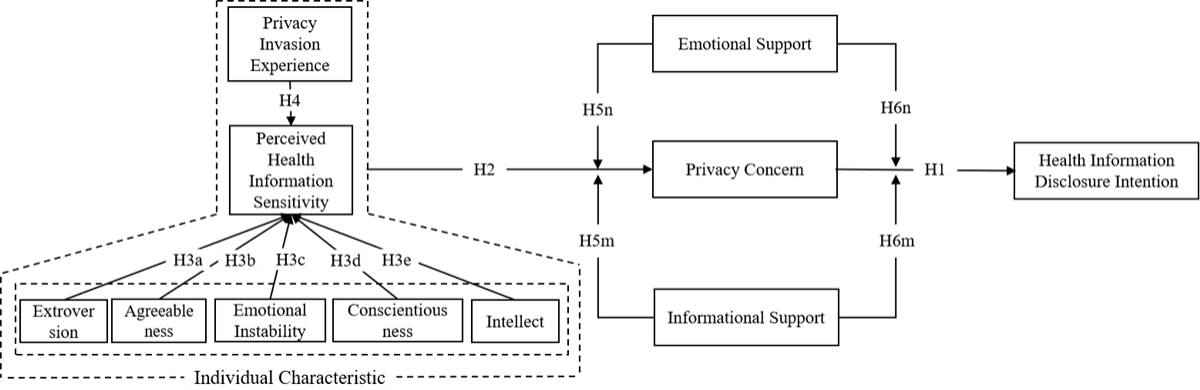
The conceptual model.

Based on the above hypotheses, this study models the impact of the interaction between benefits and costs on mHealth patients’ health information disclosure intention (Figure 1).

## Methods

### Study Approach

The overall approach was determined by the conceptual model, which sheds new light on 2 main issues: (1) the interaction effect between social support and privacy concerns regarding health information disclosure and (2) the differential impact of individual characteristics. To further analyze the conceptual model and test the proposed hypotheses, structural equation modeling (SEM) and specifically partial least square path modeling (PLS) are used. This is because PLS can analyze the complex relationships between multiple variables, and the results are reliable based on traditional factor analysis techniques [67]. A PLS analysis consists of 2 stages: all items combined weighted composites and regression analysis [68].

The data were collected using an online survey on a digital platform [69]. Participants were those who had experience using mHealth. If the candidate had not used any mHealth apps, the survey would stop; if the candidate has ever used an mHealth platform, he/she continued the survey. If the participant attempted to leave an answer blank, the questionnaire would not move on until they responded.

Many researchers agree that the determining sample size in SEM is uncertain. Schreiber et al [70] suggest that the ratio of observations to estimated parameters can be as low as 10 to 1. Thus, 110 is the minimum required sample size. We also conducted a priori sample size calculations for the minimum sample size required based on the number of latent and observed variables in the design [71]. The results showed that at a probability level of .05, a minimum size of 153 was required. Of our initial sample of 311 copies of the questionnaires retrieved, 58 were deemed unusable because they were incomplete or inconsistent. In the end, 253 valid questionnaires were obtained, and the effective rate of the questionnaire was 81.3% (253/311) . The final research model was tested by using SmartPLS version 3.0 (SmartPLS GmbH) [68], which is widely used, flexible, and efficient.

### Instrument Development and Data Collection

Before the main study, we conducted a pilot study to pretest the validity and reliability of the questionnaire before the large-scale survey. We surveyed 100 students who had used the mHealth platform, and of the resulting questionnaires, 88 were valid. Based on this pilot analysis, some of the misleading statements (cultural differences arising during the translation process) were revised. We also determined that if the factor load is less than 0.7 [72], the item would be removed. All factor loading scores were above the recommended threshold of 0.70, except for AG2, IS1, PC3, and PHI1.

The questionnaire consists of 3 parts. The first part concerns whether the candidate has had the experience of disclosing his or her health information on the website or had previously used an mHealth app. If the answer is no, his/her participation in the survey will immediately be stopped. This ensures that the questionnaire is well targeted and the data source is reliable. The second part introduces mHealth. The third part mainly gathers the respondents’ demographic information and past health history. In order to ensure construct validity, we used questionnaire items from existing studies (Table 1) and conducted reliability and validity checks (Multimedia Appendix 1).

**Table 1:** Measurement scales.

Domain and code		Items	Source	
**Health Information Disclosure Intention**			[73]	
	HID1	I am very likely to disclose my health information online.		
	HID2	I feel good that the website uses my health information.		
	HID3	It is okay to share my personal information with the health care platform.		
	HID4	I do not feel uncomfortable about sharing my personal information with the health care platform.		
**Privacy Concern**			[30]	
	PC1	I am concerned about the potential loss caused by privacy invasion.		
	PC2	I worry that others may view my personal health information.		
	PC3	Compared with other subjects on my mind, PHI^a^ is essential.		
	PC4	Compared to others (e.g., friends, relatives, and colleagues), I am more sensitive about the way websites handle my PHI.		
**Personality: Extroversion**			[74]	
	EX1	Being the center of attention.		
	EX2	Talking to a lot of different people at parties.		
	EX3	Not talking a lot.		
**Personality: Intellect**			[74]	
	IN1	Having a vivid imagination.		
	IN2	Having excellent ideas.		
	IN3	Being quick to understand things.		
**Personality:** **Conscientiousness**				[74]
	CO1	Not paying attention to details.		
	CO2	Being always ready for the future.		
	CO3	Having a rigorous work attitude.		
**Personality:** **Neuroticism**			[74]	
	EI1	Worrying about things.		
	EI2	Changing my moods frequently		
	EI3	Being easily irritated.		
**Personality:** **Agreeableness**			[74]	
	AG1	Sympathizing with the feelings of others.		
	AG2	I am always glad to help others.		
	AG3	Making people feel at ease.		
**Experience of Privacy Invasion (When it comes to the privacy invasion of health information online, your feeling can be described as)**			[73]	
	EPI1	Definitely victimized		
	EPI2	Definitely bad experiences		
	EPI3	Definitely feeling an invasion of privacy		
**Perceived Disease Severity**			[73]	
	DS1	I seldom experience major pain and discomfort for an extended period of time.		
	DS2	When it comes to chronic conditions, I believe that my condition is severe.		
	DS3	In general, I believe that the state of my health is excellent.		
**Emotional Support**			[63]	
	ENS1	When faced with health difficulties, some people sympathize with me.		
	ENS2	When faced with health difficulties, some people comfort and encourage me.		
	ENS3	When faced with health difficulties, some people pay attention to my private feelings.		
	ENS4	When faced with health difficulties, some people show interest and concern about my well‑being.		
**Informational Support**			[63]	
	INS1	When faced with health difficulties, some people do offer suggestions when I need help.		
	INS2	When faced with health difficulties, some people on online health community (mHealth) would provide information to help me overcome my problems.		
	INS3	When faced with health difficulties, some people on online health community (mHealth) would help me to discover the cause of my difficulties and provide me with suggestions		
**Perceived Health Information Sensitivity (What level of perceived health information sensitivity do I have)**			[73]	
	IS1	Medication		
	IS2	State of my health at present		
	IS3	Fitness at present		
	IS4	Medical history		

^a^PHI: personal health information.

### Data Statistics

By collecting and comparing the survey data, we found that people aged 30-49 accounted for more than 50% of respondents (148/253, 58.4%). From the perspective of educational experience, participants with a college education or above accounted for more than 90% of the total number of participants (241/253, 95.2%). Most participants had used the internet for more than 5 years and have had experience with mHealth. The demographics of participants are generally representative of the mHealth user population. These characteristics are also consistent with previous studies [75], indicating that the sample we collected is relatively comprehensive and representative. See Table 2 for demographic statistics. Those who used mHealth apps were more likely to be younger and have more education compared with those who have never used mobile apps or health apps [76]. This may because mHealth is a relatively new IT, and new IT is more easily adopted by younger and well-educated users. The data statistics show that the sample for our study is quite representative.

**Table 2:** Demographics of questionnaire respondents.

Variable and its definition		Number of samples, n	Sample frequency, %
**Age, years**			
	Less than 20 years old	1	0.51
	21-30	104	44.1
	31-40	127	49.74
	41-50	21	5.64
	More than 51	0	0.00
**Education**			
	Primary school	0	0.00
	Secondary school	12	2.05
	College degree	25	8.72
	University degree	162	72.82
	Graduate degree	41	13.85
	Postgraduate level	13	2.56
**Internet experience, years**			
	<1	0	0.00
	2-4	21	8.21
	5-7	64	25.13
	8-10	93	34.87
	>10	75	31.79
**Mobile health experience, years**			
	<1	52	18.97
	2-3	131	56.92
	4-5	53	20.51
	6-7	16	3.08
	>7	1	0.51
**Annual frequency of illness**			
	Less than 1	50	17.95
	2-3 times	136	59.49
	4-6 times	56	18.97
	7-10 times	11	3.08
	More than 10 times	1	0.51

## Results

Based on the reliability and validity of the questionnaire described above, we tested the hypothesis by structural modeling. The validation process was performed in 2 steps: one for the basic model (Table 3) and the other for the moderation effects (Table 4).

**Figure 2:**
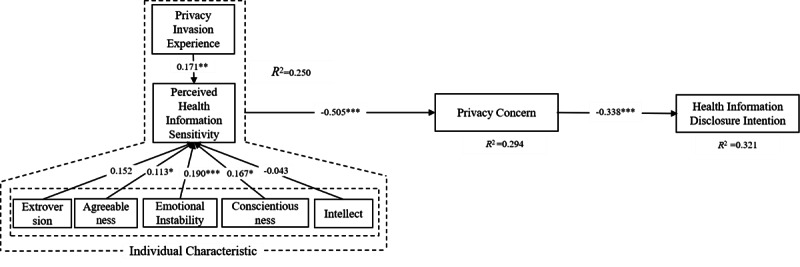
The estimated results of the basic model. Note: *** is significant at the .01 confidence level; ** is significant at the .05 confidence level; * is significant at the .1 confidence level.

The beta path coefficients show that the direct effect of perceived health information sensitivity positively affects individuals’ privacy concerns (beta path coefficient 0.505, *P*<.001), and higher privacy concern levels decrease the health information disclosure intention. Various aspects of personal dispositions influence perceived health information sensitivity in different ways. Agreeableness, emotional instability, and conscientiousness positively enhance perceived health information sensitivity, as hypothesized. However, this is not the case with extroversion and intellect. Previous online privacy invasion has a significant impact on perceived health information sensitivity (beta path coefficient 0.171, *P*=.033), as hypothesized. Prior experience with the website also has a positive influence on trust and on the intention to disclose health information online (Figure 2). Age, education level, digital literacy (mobile usage and mHealth usage), and illness frequency were controlled for in our SEM regression model. These demographics have been identified in the existing literature as determinants of privacy concerns [77,78].

**Table 3:** Main results.

Hypothesis		Path coefficients	t_460_ value^a^	*P* value	*R* ^2^
**H1**		–0.338	6.306	<.001	0.321
	Age	0.125	1.828	.068	
	Education	0.047	0.624	.533	
	Mobile usage	–0.062	0.765	.445	
	mHealth^b^ usage	0.236	2.533	.012	
	Illness frequency	–0.745	0.895	.372	
**H2**		0.505	7.851	<.001	0.294
	Age	–0.087	1.377	.169	
	Education	0.007	0.112	.911	
	Mobile usage	0.099	1.644	.101	
	mHealth usage	–0.177	2.650	.008	
	Illness frequency	–0.564	1.896	.060	
**H3**					0.173
	H3a	0.152	0.881	.379	
	H3b	0.113	1.696	.090	
	H3c	0.190	2.563	.011	
	H3d	0.167	1.733	.084	
	H3e	–0.043	0.404	.686	
**H4**		0.171	2.134	.033	0.173
	Age	–0.125	1.704	.089	
	Education	0.086	1.156	.248	
	Mobile usage	0.072	0.897	.370	
	mHealth usage	–0.095	1.258	.209	
	Illness frequency	–1.479	1.534	.127	

^a^One-sample test for null hypothesis and 2-tailed.

^b^mHealth: mobile health.

**Table 4:** Results of the moderation model.

Hypothesis			Coefficients	t_480_ value^a^	*P* value
H1			–0.287	4.927	<.001
**H5m**			–0.105	1.686	.092
	Age		0.058	1.312	.190
	Education		0.076	1.185	.236
	Mobile usage		–0.103	1.488	.137
	mHealth^b^ usage		0.180	2.623	.009
	Illness frequency		–0.258	1.335	.184
	*R* ^2^		0.337		
H1			–0.285	4.830	<.001
**H5n**			–0.060	0.891	.373
	Age		0.058	1.312	.190
	Education		0.076	1.185	.236
	Mobile usage		–0.103	1.488	.137
	mHealth usage		0.180	2.623	.009
	Illness frequency		–8.256	1.326	.187
	*R* ^2^		0.325		
H2			0.483	6.926	<.001
**H6m**			–0.171	1.690	.092
		Age	0.095	1.456	.146
		Education	0.068	1.038	.300
		Mobile usage	–0.109	1.508	.132
		mHealth usage	0.180	2.646	.008
		Illness frequency	–2.756	2.358	.197
		*R* ^2^	0.324		
H2			0.489	7.108	<.001
**H6n**			–0.137	1.455	.146
		Age	–0.094	1.568	.117
		Education	0.023	0.393	.694
		Mobile usage	0.104	1.738	.083
		mHealth usage	–0.190	2.728	.007
		Illness frequency	–4.958	1.449	.149
		*R* ^2^	0.314		

^a^One-sample test for null hypothesis and 2-tailed.

^b^mHealth: mobile health.

**Figure 3:**
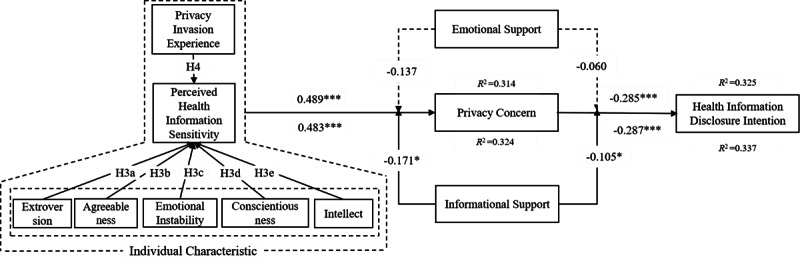
The estimated model with moderation effect. Note: *** is significant at the .01 confidence level; ** is significant at the .05 confidence level; * is significant at the .1 confidence level.

The moderation effect model mainly included 2 moderation variables of the original basic model: the emotional support and the informational support obtained through mHealth (Table 4). We find that informational support reduces the positive effect between perceived health information sensitivity and privacy concerns (beta path coefficient –0.171, *P*=.092). Furthermore, informational support will have a negative moderation effect on the relationship between privacy concerns and health information disclosure (beta path coefficient –0.105, *P*=.092). Although the *P* value is at the .1 level, the negative effect of information support on the privacy concerns is a valuable finding that supports the hypotheses [79]. However, we find that emotional support has no direct moderation effect on either privacy concerns or health information disclosure intention. Our summary in Figure 3 shows that our original hypotheses are generally supported (H1a and H1e are not significantly supported; beta path coefficient 0.152, *P*=.379 and–0.043, *P*=.686, respectively), and the moderation effects of emotional support [H5n and H6n] are also not significantly supported (beta path coefficient 0.060, *P*=.373 and –0.137, *P*=.146, respectively) in this situation.

In summary, all the paths are significant at levels greater than .1. The model fits (*R*^2^) show an acceptable level of explanatory power. As much as 9 of the 12 hypotheses are supported, with the exceptions being H3a, H3e, and H6n (Table 5).

**Table 5:** Summary of results.

Hypotheses	Content	Results
H1	Privacy concern has a negative impact on health information disclosure intention.	S^a^
H2	Higher perceived health information sensitivity is positively associated with privacy concern.	S
H3a	Extroversion has a negative impact on perceived health information sensitivity.	NS^b^
H3b	Agreeableness has a positive impact on perceived health information sensitivity.	S
H3c	Emotional instability has a positive impact on perceived health information sensitivity.	S
H3d	Conscientiousness has a positive impact on perceived health information sensitivity.	S
H3e	Intellect has a negative impact on perceived health information sensitivity.	NS
H4	Experience of privacy invasion is positively associated with perceived health information sensitivity.	S
H5m	Informational support has a negative moderation effect on the relationship between perceived health information sensitivity and privacy concern.	S
H5n	Emotional support has a negative moderation effect on the relationship between perceived health information sensitivity and privacy concern.	NS
H6m	Informational support has a negative moderation effect on privacy concern and online health information disclosure intentions.	S
H6n	Emotional support has a negative moderation effect on privacy concern and online health information disclosure intentions.	NS

^a^S: supported.

^b^NS: not supported.

## Discussion

### Principal Findings

This study empirically analyzes mHealth patients’ intentions to disclose health information. Two interesting perspectives are studied: the effect of individual characteristics and the moderation effect of benefits and costs.

### Effect of Individual Characteristics

Three relevant features (health information privacy sensitivity, personality traits, and prior privacy invasion experience) of individual characteristics are considered. Specifically, personality affects health information sensitivity in different ways. Hypotheses related to emotional instability are strongly supported, which indicates that those who are emotionally unstable tend to be nervous and frightened to disclose their medical information. Conscientious people are also likely to have higher medical information sensitivity. This may be because people with these personality traits have a higher level of risk awareness than others and tend to identify potential risks and possible negative results. In addition, previous privacy violation experiences enhance individuals’ information sensitivity and increase the negative utility of privacy concerns. In other words, people who have experienced malicious violations of personal privacy (regardless of whether this is related to health information or other information) will be extremely cautious. These results are consistent with well-known studies in the information systems field, such as Junglas et al’s [80] research in the context of location-based services, Sumner et al’s [81] study of Facebook activity, and also Abdelhamid et al’s [29] research on sharing personal health information.

One unexpected finding was that the personality factors we hypothesized would have a negative effect on perceived health information sensitivity (ie, extroversion and intellect) had no significant effect (beta path coefficient 0.152, *P*=.379 and –0.043, *P*=.686, respectively). Our results show that although extroverts are more open and prefer to disclose private information face-to-face, they are evidently less active in mHealth. Similarly, there is no significant effect between intellect and perceived health information sensitivity (beta path coefficient –0.043, *P*=.686). The finding that personality traits linked to different communication styles may have different effects is a remarkable result. It may be explained by the fact that if privacy is violated, online information can easily become widespread, which can easily have social consequences [82]. It is possible that for patients with personalities driven by intellect or extroversion, these risks make it especially difficult to reduce perceived health information sensitivity. Previous studies have yielded similar findings [83,84]. For instance, work by Bansal et al [73] indicates that a person with a high intellect trait has a strong sense of control when faced with a privacy crisis.

### Interaction Effect of Social Support

Social support has been assumed to be an important factor in improving the acceptance of telemedicine, but this assumption needs more empirical testing [85]. To fill this research gap, our results prove that in mHealth, social support can be regarded as a disutility reducer. That is, on the one hand, it alleviates patients’ privacy concerns; on the other hand, it also reduces the negative impact of privacy concerns on information disclosure intention.

Our empirical results indicate that informational support has a negative moderation effect on the relationship between privacy concern and health information disclosure intention, and it has a negative moderation effect on the relationship between health information sensitivity and privacy concerns. In other words, useful informational support encourages users to actively participate in sharing personal health information, reducing psychological and mental barriers to privacy concerns.

On the contrary, online emotional support has no significant moderation effect on privacy concerns and health information disclosure intention (beta path coefficient –0.060, *P*=.373). In other words, users will not share their information even if they receive some emotional encouragement. The literature has already proven the important role of emotional support in disease recovery [86], and it is a motivator for using mHealth [87]. However, the benefits of emotional support are not enough for it to change users’ health information sharing behaviors in mHealth. This may be explained by studies that show that people trust systems more than people [88]. In other words, it is considered safer to disclose shared health information to a system for informational support than to share the health information with other patients for emotional support.

### Theoretical and Practical Implications

Several studies have found that privacy concerns (as disclosure cost) and social support (as the benefit) have a significant impact on information disclosure intention [28,89,90]. However, the interaction between costs and benefits has received little attention. This study set out to assess the importance of the interaction effect in the context of mHealth. This research addresses the following 2 areas: (1) The interaction effect between the cost (privacy concerns) and benefits (social support) on health information disclosure intention and (2) the role of emotional support, which has been assumed to play a critical role in mHealth [18]. Our findings challenge this assumption, as they show informational support to have a conducive effect on health information disclosure intention, while emotional support has no significant effect (beta path coefficient –0.060, *P*=.373). Emotional support is an important benefit of participating in mHealth, and its impact is even higher than that of informational support. However, its ability to change patient’s willingness to disclose health information is limited, as it is relatively less significant than informational support (beta path coefficient –0.171, *P*=.092; –0.137, *P*=.146). Our results have important implications for mHealth providers, as they show that social support, especially informational support, is effective in prompting patients using mHealth to share their health information. However, emotional support has not been fully utilized, and its role in mHealth therefore needs more attention.

### Limitations and Future Research Directions

There are several limitations to our study. First, according to the previous literature, there are several variables that may be related to patients’ privacy concern, such as personality tendencies, current physical conditions, context of use (eg, different health status, locations, and times), and so on. However, we limited our variables to 3 that have been widely studied in the literature (ie, personality, experience of privacy invasion, and perception of sensitive health information) in order to focus on the moderation effects between privacy concern and social support. In future studies, more variables can be included in our model, which would result in broader insight into online health information disclosure.

Second, mHealth platforms in China have been specially designed to incorporate social support in such a way that it can be provided from both professional doctors’ and patients’ perspectives, which thus allows this paper to obtain a unique data set on social support. However, the literature has already shown that culture plays an important role in privacy calculus, especially in the way in which culture and individual characteristics combine to influence patients’ perceptions and decisions [91]. Therefore, in future studies, testing this model within different cultures would illuminate how the results are influenced by cultural differences. It is also worth further studying whether the platform’s social support function, which is specifically designed for the Chinese cultural context (in which government policies support doctors in providing online consultations and social support) can serve as a reference for the development of mHealth platforms worldwide.

Third, the mechanisms of online emotional support in an mHealth context should be studied. Our results indicate that while informational support plays a significant role in addressing privacy concerns, emotional support has less effectiveness. Emotional support can enhance engagement in online communities [59], but in the face of privacy concerns, this finding may be invalid because emotional support fails to establish trust on health information. Future studies may explore how trust affects emotional support and health information disclosure in mHealth.

### Conclusion

In this paper, we used an SEM approach to study patients’ health information disclosure intentions in an mHealth context. While most studies to date have considered disclosure costs and benefits independently, we simulated patients’ online disclosure behaviors by examining the interactive effects of privacy concerns and social support. Individual characteristics were also included in our model to help us reach a further understanding of the combined effects. Our results offer several insights into the driving forces behind patients’ disclosure intentions related to health information, and they demonstrate the usefulness and value of mHealth. Finally, this study may stimulate additional research to further enrich the understanding of health information disclosure in mHealth.

## Appendix

Multimedia Appendix 1 Reliability and Validity.

## Abbreviations

mHealth: mobile health
PCT: privacy calculus theory
PLS: partial least square path modeling
SEM: structural equation modeling
