# Supplementary material for: Privacy Concerns About Health Information Disclosure in Mobile Health: Questionnaire Study Investigating the Moderation Effect of Social Support
Source: JMIR Mhealth Uhealth. 2021 Feb 8;9(2):e19594. doi: 10.2196/19594 (PMC7899802; doi:10.2196/19594)
Supplement: Multimedia Appendix 1 [file mhealth_v9i2e19594_app1.docx]

## Appendix A

#### Reliability and Validity

In combination with the pilot analysis, we tested the overall reliability (KOM and Bartlett’s Test) and item reliability (item loading) of our study. Then we examined the convergent validity (Cronbach’s alpha (CA), composite reliability (CR) and the Average Variance Extracted (AVE)). Based on the pilot analysis results, we chose 11 latent variables and included 37 items in the questionnaire to measure the disruptions and performance efficiency in order to understand whether the questions in our questionnaire are all reliable. We then measured the reliability of the items in the questionnaire.

The reliability test shows, first, the overall reliability of our questionnaire by demonstrating that it measures in a way that is internally consistent across different respondents. KMO and Bartlett’s test were used to measure suitability of the item data for the factor analysis, based on correlations and covariance matrixes. For the KMO, a value of 0.8 to 1 is acceptable value [1], and the KMO value in our data is 0.893.

To assess the quality of the proposed measurement model, we start with the item loading. It shows the reliability of the items associated with a particular construct, and to ensure the quality, the respective loading should exceed the suggested threshold value of 0.7 (see Table A1). Then we examine convergent validity, which is the extent to which the observable variables actually measure a conceptualized construct. To examine the convergent validity of measurement constructs, we use Cronbach’s alpha (CA) and composite reliability (CR). The results extracted for the constructs in Table A1 meet the proposed threshold of 0.7, which is appropriate and considered adequate to demonstrate internal consistency [2]. Discriminant validity was tested using the square root of AVE values for each construct. The values in Tables A2 and A3 show that the loading value on the construct is significantly larger than cross factor loading [3].

Table A1. Results of Confirmatory Factor Analysis

| **Construct** | **Item** | **Factor Loadings** | **Cronbach's α** | **CFR** | **AVE** |
| --- | --- | --- | --- | --- | --- |
| Health Information Disclosure Intention | HID2 | 0.866 | 0.828 | 0.897 | 0.744 |
|  | HID3 | 0.864 |  |  |  |
|  | HID4 | 0.858 |  |  |  |
| Privacy Concern | PC1 | 0.942 | 0.922 | 0.951 | 0.865 |
|  | PC2 | 0.909 |  |  |  |
|  | PC4 | 0.939 |  |  |  |
| Perceived Health Information Sensitivity | IS2 | 0.847 | 0.851 | 0.909 | 0.770 |
|  | IS3 | 0.911 |  |  |  |
|  | IS4 | 0.873 |  |  |  |
| Experience of Privacy Invasion | EPI1 | 0.938 | 0.929 | 0.955 | 0.876 |
|  | EPI2 | 0.934 |  |  |  |
|  | EPI3 | 0.936 |  |  |  |
| Extroversion | EX1 | 0.995 | 0.906 | 0.857 | 0.671 |
|  | EX2 | 0.738 |  |  |  |
|  | EX3 | 0.692 |  |  |  |
| Agreeableness | AG1 | 0.971 | 0.728 | 0.858 | 0.754 |
|  | AG3 | 0.753 |  |  |  |
| Emotional Instability | EI1 | 0.906 | 0.892 | 0.931 | 0.818 |
|  | EI2 | 0.948 |  |  |  |
|  | EI3 | 0.857 |  |  |  |
| Conscientiousness | CO1 | 0.906 | 0.774 | 0.865 | 0.682 |
|  | CO2 | 0.773 |  |  |  |
|  | CO3 | 0.792 |  |  |  |
| Intellect | IN1 | 0.968 | 0.847 | 0.869 | 0.692 |
|  | IN2 | 0.727 |  |  |  |
|  | IN3 | 0.781 |  |  |  |
| Emotional Support | ENS1 | 0.807 | 0.817 | 0.878 | 0.643 |
|  | ENS2 | 0.819 |  |  |  |
|  | ENS3 | 0.770 |  |  |  |
|  | ENS4 | 0.811 |  |  |  |
| Informational Support | INS1 | 0.797 | 0.758 | 0.861 | 0.674 |
|  | INS2 | 0.846 |  |  |  |
|  | INS3 | 0.819 |  |  |  |

Table A2 Item Loadings and Cross-Loadings

|  | **AG** | **CO** | **EI** | **EX** | **IN** | **IS** | **OES** | **OIS** | **PC** | **PHI** | **PPI** |
| --- | --- | --- | --- | --- | --- | --- | --- | --- | --- | --- | --- |
| **AG1** | **0.971** | 0.480 | 0.024 | 0.163 | 0.384 | 0.119 | 0.333 | 0.372 | 0.102 | 0.178 | 0.064 |
| **AG3** | **0.753** | 0.511 | 0.013 | 0.312 | 0.514 | 0.044 | 0.348 | 0.439 | 0.019 | 0.309 | -0.059 |
| **CO1** | 0.417 | **0.906** | -0.096 | 0.307 | 0.471 | 0.172 | 0.424 | 0.529 | 0.075 | 0.284 | -0.033 |
| **CO2** | 0.445 | **0.773** | -0.173 | 0.310 | 0.410 | 0.096 | 0.418 | 0.530 | 0.080 | 0.318 | 0.029 |
| **CO3** | 0.507 | **0.792** | -0.143 | 0.290 | 0.500 | 0.102 | 0.453 | 0.539 | 0.094 | 0.343 | -0.028 |
| **EI1** | 0.060 | -0.070 | **0.906** | -0.142 | -0.114 | 0.237 | -0.136 | -0.072 | 0.438 | -0.140 | 0.317 |
| **EI2** | -0.010 | -0.176 | **0.949** | -0.163 | -0.150 | 0.225 | -0.144 | -0.149 | 0.390 | -0.102 | 0.281 |
| **EI3** | 0.004 | -0.202 | **0.857** | -0.126 | -0.170 | 0.136 | -0.087 | -0.156 | 0.282 | 0.011 | 0.236 |
| **EX1** | 0.231 | 0.373 | -0.186 | **0.995** | 0.501 | 0.115 | 0.340 | 0.189 | -0.065 | 0.419 | -0.130 |
| **EX2** | 0.265 | 0.422 | -0.295 | **0.738** | 0.536 | -0.002 | 0.336 | 0.302 | -0.093 | 0.363 | 0.017 |
| **EX3** | 0.222 | 0.340 | -0.301 | **0.691** | 0.494 | -0.014 | 0.340 | 0.241 | -0.096 | 0.325 | -0.079 |
| **IN1** | 0.437 | 0.505 | -0.146 | 0.474 | **0.969** | 0.075 | 0.356 | 0.380 | -0.018 | 0.345 | -0.037 |
| **IN2** | 0.320 | 0.422 | -0.194 | 0.426 | **0.729** | -0.009 | 0.264 | 0.272 | -0.041 | 0.240 | -0.048 |
| **IN3** | 0.375 | 0.514 | -0.153 | 0.375 | **0.781** | 0.028 | 0.302 | 0.370 | -0.016 | 0.165 | -0.078 |
| **IS2** | 0.031 | 0.072 | 0.186 | 0.105 | 0.069 | **0.847** | 0.025 | 0.080 | 0.366 | -0.177 | 0.215 |
| **IS3** | 0.175 | 0.209 | 0.206 | 0.094 | 0.045 | **0.911** | 0.172 | 0.213 | 0.499 | -0.133 | 0.182 |
| **IS4** | 0.063 | 0.121 | 0.212 | 0.144 | 0.081 | **0.873** | 0.064 | 0.088 | 0.448 | -0.116 | 0.204 |
| **OES1** | 0.285 | 0.403 | -0.094 | 0.234 | 0.310 | 0.080 | **0.807** | 0.550 | -0.002 | 0.361 | -0.102 |
| **OES2** | 0.319 | 0.433 | -0.085 | 0.243 | 0.284 | 0.042 | **0.819** | 0.555 | 0.062 | 0.304 | -0.076 |
| **OES3** | 0.241 | 0.379 | -0.113 | 0.214 | 0.262 | 0.146 | **0.770** | 0.509 | 0.092 | 0.239 | -0.019 |
| **OES4** | 0.329 | 0.426 | -0.159 | 0.344 | 0.330 | 0.086 | **0.811** | 0.520 | 0.018 | 0.334 | -0.038 |
| **OIS1** | 0.376 | 0.451 | -0.198 | 0.181 | 0.259 | 0.095 | 0.520 | **0.796** | 0.035 | 0.242 | -0.142 |
| **OIS2** | 0.400 | 0.601 | -0.032 | 0.135 | 0.326 | 0.087 | 0.551 | **0.847** | 0.022 | 0.280 | -0.151 |
| **OIS3** | 0.282 | 0.501 | -0.107 | 0.114 | 0.424 | 0.184 | 0.567 | **0.819** | 0.138 | 0.282 | -0.012 |
| **PC1** | 0.088 | 0.105 | 0.421 | -0.076 | -0.007 | 0.484 | 0.029 | 0.085 | **0.943** | -0.291 | 0.533 |
| **PC2** | 0.036 | 0.074 | 0.367 | -0.054 | 0.027 | 0.442 | 0.058 | 0.076 | **0.908** | -0.295 | 0.511 |
| **PC4** | 0.117 | 0.092 | 0.389 | -0.031 | -0.065 | 0.479 | 0.044 | 0.065 | **0.939** | -0.285 | 0.461 |
| **PHI2** | 0.262 | 0.360 | -0.060 | 0.331 | 0.308 | -0.177 | 0.367 | 0.303 | -0.231 | **0.866** | -0.183 |
| **PHI3** | 0.154 | 0.285 | -0.073 | 0.389 | 0.214 | -0.134 | 0.340 | 0.248 | -0.283 | **0.864** | -0.234 |
| **PHI4** | 0.189 | 0.308 | -0.127 | 0.354 | 0.311 | -0.100 | 0.311 | 0.297 | -0.293 | **0.858** | -0.158 |
| **PPI1** | 0.061 | 0.003 | 0.297 | -0.089 | -0.010 | 0.228 | -0.038 | -0.101 | 0.509 | -0.184 | **0.938** |
| **PPI2** | 0.014 | -0.039 | 0.276 | -0.132 | -0.074 | 0.190 | -0.098 | -0.128 | 0.472 | -0.186 | **0.934** |
| **PPI3** | 0.016 | -0.019 | 0.304 | -0.160 | -0.065 | 0.214 | -0.087 | -0.114 | 0.530 | -0.253 | **0.936** |

Table A3 Results of discriminant Validity Testing

|  | **AG** | **CO** | **EI** | **EX** | **IN** | **IS** | **ENS** | **INS** | **PC** | **HID** | **EPI** |
| --- | --- | --- | --- | --- | --- | --- | --- | --- | --- | --- | --- |
| **AG** | **0.869** |  |  |  |  |  |  |  |  |  |  |
| **CO** | 0.535 | **0.826** |  |  |  |  |  |  |  |  |  |
| **EI** | 0.023 | -0.154 | **0.904** |  |  |  |  |  |  |  |  |
| **EX** | 0.222 | 0.361 | -0.161 | **0.819** |  |  |  |  |  |  |  |
| **IN** | 0.459 | 0.552 | -0.154 | 0.480 | **0.832** |  |  |  |  |  |  |
| **IS** | 0.109 | 0.159 | 0.230 | 0.130 | 0.073 | **0.877** |  |  |  |  |  |
| **ENS** | 0.369 | 0.513 | -0.141 | 0.326 | 0.373 | 0.106 | **0.802** |  |  |  |  |
| **INS** | 0.427 | 0.633 | -0.132 | 0.172 | 0.414 | 0.151 | 0.666 | **0.821** |  |  |  |
| **PC** | 0.087 | 0.097 | 0.422 | -0.058 | -0.017 | 0.504 | 0.046 | 0.081 | **0.930** |  |  |
| **HID** | 0.233 | 0.367 | -0.101 | 0.416 | 0.322 | -0.157 | 0.392 | 0.328 | -0.313 | **0.863** |  |
| **EPI** | 0.034 | -0.018 | 0.313 | -0.135 | -0.051 | 0.226 | -0.078 | -0.121 | 0.539 | -0.222 | **0.936** |

^a^Note: The diagonal data is the square root of the arithmetic of the corresponding constructed AVEs.

**References**

1. Amin N, Chipika J. A factor-analytic approach to peasant differentiation and household food security in Zimbabwe. 1994.

2. Churchill Jr GA. A paradigm for developing better measures of marketing constructs. Journal of marketing research. 1979;16(1):64-73.

3. Chin WW, Marcolin BL, Newsted PR. A partial least squares latent variable modeling approach for measuring interaction effects: Results from a Monte Carlo simulation study and an electronic-mail emotion/adoption study. Information systems research. 2003;14(2):189-217.
